# Section 2. Exercise-Induced Bronchospasm: Albuterol versus Montelukast: *Highlights of the Asthma Summit 2009: Beyond the Guidelines*

**DOI:** 10.1097/WOX.0b013e3181d25eac

**Published:** 2010-02-15

**Authors:** Gene Colice, William J Calhoun

**Affiliations:** 1 Pulmonary, Critical Care and Respiratory Services, Washington Hospital Center, Professor of Medicine, The George Washington University School of Medicine, Washington, DC; 2 Department of Internal Medicine, University of Texas Medical Branch, Galveston, TX

**Keywords:** asthma, exercise-induced bronchospasm, exercise-induced asthma, airway hyperreactivity, bronchial hyperresponsiveness, albuterol, montelukast

## Abstract

Exercise-induced bronchospasm (EIB) involves airway obstruction with an onset shortly after exercising. It can occur in individuals without a diagnosis of asthma, but is most common in asthmatic patients (and in this scenario may be referred to as exercise-induced asthma, EIA), correlating with the patient's degree of airway hyperreactivity. While albuterol is the most commonly used rescue and prophylactic medication for EIB, the leukotriene antagonist, monetlukast, may be an appropriate choice for some patients. Clinical data have shown that once-daily treatment with montelukast (5 or 10 mg tablet) can offer protection against EIB within 3 days for some patients. Such an approach might be preferred for patients who have difficulty with inhaled medications and for children who cannot access their inhalers during the school day. Montelukast also may be an option to reduce side effects associated with albuterol for individuals who exercise regularly.

## Albuterol rather than montelukast: commentary by gene colice, MD, FCCP

Exercise-induced bronchospasm (EIB) commonly occurs in a wide range of patients. It can affect up to 70 to 80% of patients with symptomatic asthma. The magnitude of the bronchoconstriction with EIB in asthma patients appears to correlate with their degree of bronchial hyperresponsiveness (BHR) [1]. EIB also appears in patients who do not have asthma, but nonetheless exhibit BHR. In a study of 281 nonasthmatic children, 10 (4%) experienced EIB and the EIB was significantly associated with BHR to histamine [2]. However, neither asthma nor BHR are required for the occurrence of EIB. In members of the 1998 US Winter Olympic team, EIB occurred in up to 50% of the athletes, with the specific proportion varying by sport [3].

Before considering which treatments are preferable for EIB, one must understand what EIB is not. It is not shortness of breath during exercise in patients with asthma. Asthma patients who get short of breath while exercising usually have uncontrolled disease, not EIB. EIB should also not be considered exercise-induced *asthma *(also referred to as *EIA*). Unlike allergen exposure and occupational sensitization, exercise itself does not induce asthma.

**Figure 1:**
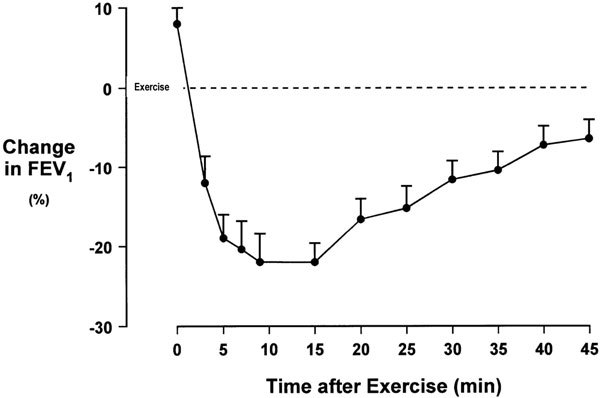
**EIB as demonstrated by the decline in FEV_1_**. Figure provided by Gene Colice, MD.

EIB is a distinct and well-defined sequence of events associated with bronchospasm that occurs shortly after completion of exercise. Patients will describe cough, chest tightness, wheezing, and shortness of breath with EIB, similar to typical asthma symptoms. EIB is confirmed by a decline in the forced expiratory volume in the first second (FEV_1_), confirming airway obstruction (Figure 1). The intensity of the bronchoconstriction typically peaks at 5 to 10 minutes after exercise and usually remits about 60 minutes thereafter.

A peculiar feature of EIB is the so-called refractory period. If exercise is repeated within 1 to 3 hours, there will be less of an EIB response. This has led some to advocate a warm-up for asthma patients before full intensity exercise. Whether late bronchoconstriction, defined as that occurring 4 to 6 hours after exercise, is a part of EIB remains in debate. Although there are numerous theories about why EIB occurs, the underlying mechanisms of EIB are not clear.

Several drugs and some nonpharmacological approaches seem to be effective in protecting against, or relieving symptoms of, EIB. The National Asthma Education and Prevention Program Third Expert Panel Report (EPR-3) recommends use of either short-acting or long-acting inhaled *β*_2_-agonists as the preferred preventive treatment for EIB [4]. Using these agents before exercise will effectively prevent EIB in more than 80% of patients. The EPR-3 guidelines do note that leukotriene receptor antagonists (LTRA) can attenuate EIB in up to 50% of patients, but the onset of the protective effect of LTRAs occurs only hours after administration of these agents [4].

**Figure 2:**
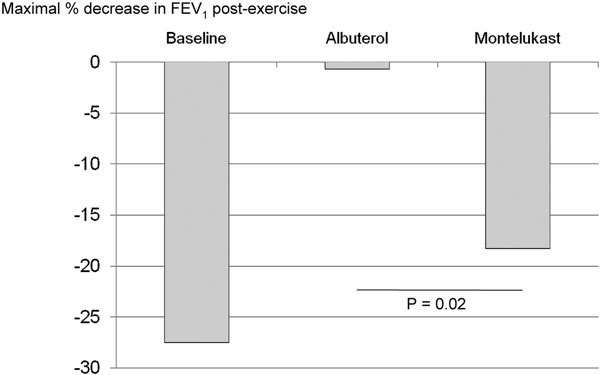
**Effect of albuterol and montelukast on the percent decrease in FEV_1 _after exercise challenge in 11 children (7-17 years) with exercise-induced asthma (defined as ≥ 15% drop in FEV_1_)**. Patients received 3-7 days of montelukast (5-10 mg/d) or 2 puffs of albuterol MDI just before exercise challenge and then were crossed over to the alternate treatment [8].

There are clear advantages of albuterol, or other short-acting inhaled *β*_2_-agonists, over montelukast in managing EIB. The protective effect of albuterol against EIB is apparent quickly, unlike LTRAs that must be given hours before exercise to prevent EIB. Albuterol can be administered 15 to 30 minutes before exercise in both children and adults to prevent exercise-related symptoms [5-9]. In addition, albuterol seems to more effectively prevent EIB than montelukast. In a direct comparative study patients with proven EIB were treated with either montelukast for 3-7 days or albuterol 15 minutes preexercise. Albuterol virtually eliminated the postexercise fall in FEV_1 _in these patients, whereas montelukast provided only a mild attenuating effect [8] (Figure 2). A preexercise warm up has also been shown to not be as effective as albuterol pretreatment for preventing EIB [5].

Albuterol can also be used as a rescue medication if symptoms of EIB occur despite use of preventive measures [9]. This is another differentiating factor between albuterol and LTRAs. Of 3 studies that evaluated the effect of single-dose montelukast in preventing EIB, 2 specified that albuterol was to be used for rescue therapy if patients became symptomatic during exercise,[10,11] and one included use of short-acting *β*-agonists as rescue therapy as a prespecified secondary end point [12]. A total of 31% of the patients in one study who used montelukast as prophylaxis experienced postexercise falls in FEV_1 _exceeding 15% [10]. This degree of bronchospasm would usually be treated with a rescue medication, specifically albuterol. Similarly, in the other study 17% of patients experienced declines of more than 20% [11]. In the third study, 6% of patients were given albuterol for rescue despite being pretreated with montelukast to prevent EIB [12]. From the practical, patient care perspective, asthma patients using montelukast to prevent EIB should still have albuterol on hand to treat unanticipated episodes of EIB that might still occur.

There are concerns about the use of albuterol in EIB. Tachyphylaxis is a concern with the regular use of albuterol, and the question remains whether using albuterol pretreatment for exercise daily results in tachyphylaxis that might make therapy less effective. In one study in which patients took albuterol or placebo for 6 to 10 days and exercised without pretreatment, the exercise-induced fall in FEV_1 _was greater among the group using albuterol than it was among the placebo group [13]. In this study, though, treatment with albuterol after EIB developed effectively improved lung function. Similar results were seen in another study where patients took albuterol 4 times a day for 7 days [9]. However, in this study albuterol administered immediately before exercise was still effective in preventing EIB [9]. It should be emphasized, though, the guidelines on the management of asthma make it clear that regular use of albuterol is not recommended [4].

Tachyphylaxis to the effects of albuterol might also occur if patients were regularly using an inhaled long-acting *β*_2_-agonist. Regular use of salmeterol has been reported to reduce the effect of albuterol treatment to protect against methacholine-induced bronchoconstriction [14]. Nelson et al, though, showed that the acute bronchodilator response to albuterol was maintained in asthma patients who were either inhaled corticosteroid (ICS)-naive or receiving ICS, regardless of regular salmeterol use [15]. However, another study found that the effect of albuterol against EIB was reduced in asthmatic patients who regularly used a combination of ICS and long-acting *β*-agonists [16]. Overall, it is unclear whether the regular use of an inhaled long-acting *β*_2_-agonist will reduce either the protective or treatment effect of albuterol in EIB. Any possible decreased protection by albuterol for EIB should be carefully weighed against the overall clinical benefits achieved from using combination therapy with an ICS and an inhaled long-acting *β*_2_-agonist.

An interesting concern raised by exercise physiologists, particularly relevant to high performance athletes, is the potential relationship between any cardiovascular effects of albuterol and enhanced exercise performance. However, after 2 preexercise puffs of albuterol, serum albuterol levels are well below meaningful threshold levels, and no cardiovascular effects have been detected at this dose. Furthermore, albuterol does not enhance bronchodilation during exercise and high doses of salmeterol or albuterol have not been shown to effect performance There is "compelling" evidence that inhaled *β*_2 _agonists do not enhance athletic performance in health adults [17].

In summary, albuterol is convenient to use, and the data overwhelmingly support its efficacy and safety for EIB, both as pretreatment to prevent symptoms and as rescue for acute symptoms. The EPR-3 is correct that albuterol should be the preferred treatment for EIB.

## Montelukast and exercise-induced asthma: commentary by William J. Calhoun, MD, FACP, FCCP, FAAAAI, FACAAI

EIA is defined as bronchospasm triggered by exercise in individuals who have asthma. There is controversy over using the term EIA versus EIB. However, EIA is an important clinical phenotype that continues to be a topic for controversy and debate. The mechanisms underlying EIA, particularly the involvement of mediator release and the importance of neural and local control, are not known. In addition, whether EIA involves a vascular response or an inflammatory one (or both) is not clear. These questions must be answered to facilitate identification of the best approaches for preventing EIA.

**Figure 3:**
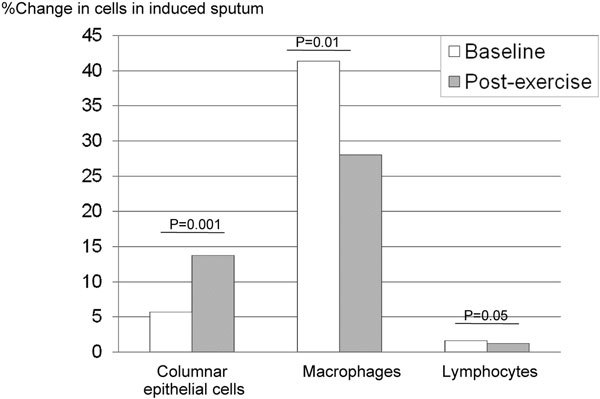
**Changes in inflammatory cells in induced sputum after exercise challenge in 25 patients with asthma and EIB**. No significant changes were observed in eosinophils or neutrophils [20].

**Figure 4:**
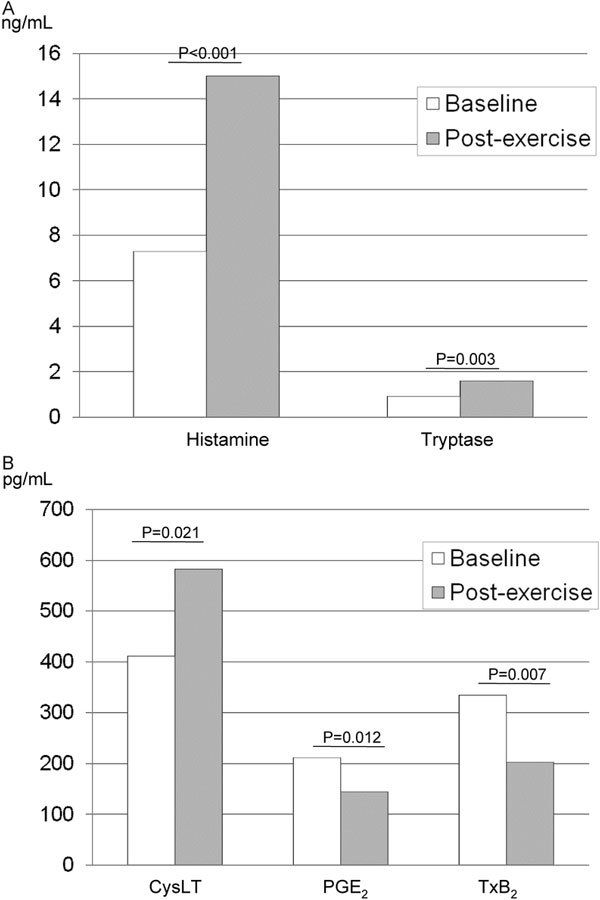
**Effects of exercise challenge on the levels of mast cell mediators (A) and eicosanoids (B) in induced sputum from 25 patients with asthma and EIB**. CysLT, cysteinyl leukotrienes; PGE_2_, prostaglandin E_2_; TxB_2_, thromboxane B_2 _[20].

There is some evidence of a link between airway inflammation and/or airway injury and EIA. Early bronchoalveolar lavage studies found no evidence that inflammatory mediators were released in EIA to the degree seen with viral infection or allergen challenge,[18,19] but later data including replication of these studies showed evidence of some release of eicosanoids, including cysteinyl leukotrienes [20]. A sputum-induction study reported that infiltrating inflammatory cells typical for asthma, such as macrophages, lymphocytes, eosinophils, and neutrophils, did not change with EIA (Figure 3) [20]. However, the number of epithelial cells increased to a significant degree, and this increase correlated with the maximum fall in FEV_1 _[20]. This study also confirmed earlier observations showing increased levels of cysteinyl leukotrienes during EIA, and tryptase and histamine (Figure 4). Based on these findings, inhibiting cysteinyl leukotrienes might be an effective strategy for managing EIB.

**Figure 5:**
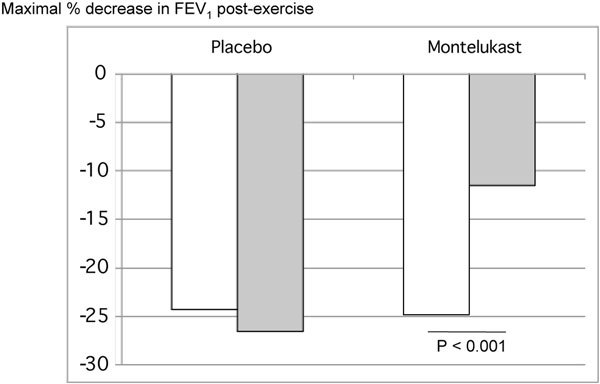
**Effect of treatment on the percent decrease in FEV_1 _after exercise challenge in 40 children (6-18 years) with exercise-induced asthma (defined as ≥ 15% drop in FEV_1_)**. Patients received 4 weeks of montelukast (5-10 mg/d) or placebo [21].

Taken on a regular once-daily basis, the LTRA montelukast has been shown to improve the exercise-induced deficit in lung function: blunting the early fall in FEV_1_, reducing the area under the curve (AUC), and decreasing the time to recovery of normal lung function (Figure 5) [21-26].

Similarly, montelukast has been demonstrated to reduce the fall in FEV_1 _after eucapnic voluntary hyperventilation, an experimental model of EIB that focuses on the cooling and drying of the airway [23]. The benefit of montelukast against exercise challenge can be seen as early as 3 days on regular treatment [22]. Specific challenge studies have reported improvements in lung function as soon as 2 hours after a single dose [10,11]. The positive effects of montelukast therapy can persist for up to 8 weeks after discontinuation,[24] suggesting that treatment induces physiological changes in the airway. No tachyphylaxis or loss of protection have been observed [21,22,24,26].

For the most part, montelukast provides comparable (and sometimes even superior) protection to other approaches used to address EIB. For example, in children with mild asthma both montelukast and fitness training reduced BHR and also decreased the incidence of EIB by 50% [27]. Another randomized study in atopic children reported a 54% reduction in the maximum postexercise fall in FEV_1 _with montelukast (5 or 10 mg once daily) compared with reductions of 52, 32, and 24% for montelukast budesonide (100 *μ*g bid), budesonide alone, and budesonide formoterol (100 *μ*g/4.5 *μ*g bid), respectively; patients treated with placebo showed an increase of 9% [21]. In a study in adults with EIA single doses of montelukast (10 mg) and salmeterol (42 *μ*g) provided comparable prophylaxis against EIB more than 12 hours, reducing the fall in FEV_1 _by ~70% (*P *< 0.001 for salmeterol, and *P *< 0.001 for montelukast) [28]. The difference between the 2 agents was an onset of action within 10 minutes after challenge for salmeterol compared with an onset within 1 hour for montelukast. A comparison of the effects of regular use of salmeterol (50 *μ*g bid) and montelukast (10 mg qd) on EIB more than 8 weeks in 197 patients with mild asthma reported few differences between the 2 treatments, but overall, montelukast provided slightly better control with no tolerance evident and fewer adverse events [29].

The benefit of montelukast compared with albuterol, however, is not as clear cut. In a crossover study of 11 children (7-17 years), EIB was better controlled by pretreatment with 2 puffs of albuterol than with 3-7 days of daily treatment with montelukast (5 or 10 mg) [8]. Albuterol significantly reduced EIB in 100% of the patients, compared with 55% for montelukast. Thus, a dose of albuterol given immediately before exercise might be superior to daily dosing with montelukast. However, other data suggest that montelukast preserves the bronchodilator responsiveness to albuterol [30]. Some patients may benefit from using both, and additional study is warranted to address this.

For patients who exercise regularly and use albuterol to prevent EIB, adding montelukast may reduce the dose of albuterol needed and the associated side effects. For example, despite the efficacy of albuterol in preventing EIB, patients in one study showed a larger decrement in postexercise FEV_1 _than those who used placebo [9]. The question also remains as to whether the cardiovascular effects observed with higher doses of albuterol also occur in relation to multiple, frequent dosing. High doses of beta-agonists are associated with abnormalities in oxygen pulse in the lungs. A crossover comparison of 5 days of montelukast (10 mg) and 5 days of salmeterol (50 *μ*g bid) in 18 patients with EIB showed comparable improvements lung function and performance, but better gas exchange during exercise with montelukast [31]. Unmeasured effects, such as possible activation of counter-regulatory pathways observed with chronic *β*-receptor stimulation, also might pose a risk.

Convenience is another consideration. Some patients who only have exercise-related symptoms might prefer to use albuterol as needed immediately before exercise. Generally, these are patients who are able to carry their inhaler with them and use it when needed. However, this may not be the case for others, such as schoolchildren who are physically active throughout the day and may have limited access to their own medication. In such cases the protection offered by a regular tablet of montelukast might be preferable to pretreatment with an inhaler.

It is possible that EIB, like asthma, is mechanistically heterogeneous and that understanding that heterogeneity might provide better clues to guide prophylaxis. However, phenotypic heterogeneity is often hidden in reports of group mean data. Online repositories containing individual data could, thus, be useful. In light of the potential heterogeneity in EIB and among patients, it is untenable to propose that only one therapy is appropriate for EIB. The proven efficacy and safety profile of montelukast make this drug one of the preferred therapies for treating EIB. The final choice will depend on the patient's disease profile, lifestyle, and preferences.

## Discussion

**Dr. Calhoun: **Obviously, it is scientifically untenable for either of us to take a position that the only appropriate treatment for EIB is either montelukast or albuterol. However, I would like to emphasize three things. First, I think the question of tachyphylaxis is important. Dr. Colice presented data showing that people who had regular albuterol for a week had a larger decrement in FEV_1 _postexercise than those who were on placebo. Four times a day albuterol is not a lot, it does not saturate the beta receptors continuously; so, to the extent that patients needed more and more and more albuterol, the issue of tachyphylaxis is significant. Second, there are things that are unmeasured. For example, activation of the counter-regulatory phospholipase C pathway with chronic stimulation of the beta receptors is probably not good. Third, with regard to convenience, it certainly might be easy for some persons to use an albuterol inhaler immediately before exercise. However, that might not be the case for others, like those who exercise during the middle of the day or children who have recess or physical education during the day. For them, a pill taken in the morning might be the best option. Finally, I don't think any of us would advocate using albuterol regularly four times a day to prevent EIB. The risks of that sort of strategy outweigh the benefits in contrast to the relatively minimal risks of montelukast.

**Dr. Colice: **Yes, the data show that if a patient uses albuterol regularly, the fall in FEV_1 _with EIB might actually be enhanced. However, when these patients then took a dose of albuterol preexercise, it still completely eliminated the fall in FEV_1 _during exercise. So, while regular use of albuterol results in tachyphylaxis, whether it actually enhances EIB is unclear. Patients should not be using albuterol regularly; but, if they are doing so contrary to all recommendations, using albuterol before exercise still will be effective. And what about using a long-acting bronchodilator (LABA) regularly? There are data showing that regular use of a LABA reduces its bronchoprotective effect; but other data show that patients using a LABA still have a good bronchodilator response to a single dose of albuterol. For patients using combination therapy, the bottom line is that regular use of an ICS does not protect against tachyphylaxis to beta-agonists. However, this must be weighed against the benefit achieved from combination therapy to treat asthma. Finally, with regard to whether using albuterol for EIB in the elite athlete can enhance exercise performance and provide a competitive advantage, I think the answer to that is clearly, no. Albuterol levels are almost undetectable after two puffs, well below the nanogram threshold that the Olympic committees have designated. Also, there's certainly not enough to do anything meaningful from a systemic perspective; no relevant changes in pulse, heart rate and potassium after two puffs.

**Dr. Storms: **In terms of albuterol or salmeterol being performance enhancing, we did one study on each at the Olympic training center, looking at things like time to peak and lactate threshold. There was no effect on performance with either high-dose albuterol or high-dose salmeterol.

**Dr. Colice: **Dr. Storms, an intriguing thing about EIB to me is that it can occur in patients who have no asthma and no BHR. Do you know what is going on in these elite athletes?

**Dr. Storms: **There is an "elite athlete syndrome" that is talked about. It's noneosinophilic; it's neutrophilic, probably an airway injury syndrome. These patients respond to nothing and are a lot more likely to be positive to methacholine challenge than to exercise challenge.

**Dr. Colice: **I assume that if you can exercise to the point where the minute ventilation increases substantially, this could happen.

**Dr. Calhoun: **Yes. I think it requires an extraordinary degree of training so that the change in minute ventilation is dramatic.

**Dr. Busse: **With cross-country skiers, the temperature in their airways really drops, supporting the notion that there might be some injury which brings about these changes. It's a very unique group.

**Dr. Calhoun: **Yes, talking about exercise as a trigger of bronchospasm in asthmatics who are being treated for their asthma as compared with these types of athletes muddles the difference between EIB and EIA.

**Dr. Colice: **I don't agree.

**Dr. Calhoun: **EIB is bronchospasm which occurs not necessarily in the context of asthma. It's an important clinical question to understand what happens with exercise as a trigger of bronchospasm in patients with asthma (EIA) who may be treated with ICS and/or LABA. They may represent a different subset of patients.

**Dr. Colice: **Yes, EIB seems to occur more often in patients with asthma and in those who don't have asthma but have BHR. However, it also can occur in elite athletes who do not have asthma or BHR. Thus, EIB is a very novel clinical situation that occurs in a wide range of patients.

**Dr. Lemanske: **What does the FDA require for a pharmaceutical company to get an indication for a drug for use in EIB?

**Dr. Colice: **The Food and Drug Administration (FDA) first of all requires clear documentation of patients with EIB, which includes normal FEV_1_, no evidence of symptomatic asthma, and not using ICS. EIB must be demonstrated on 2 occasions pretrial by an exercise test, showing a greater than 15% fall in FEV_1 _usually within 3 to 5 minutes postexercise. Then you have to show significant protection with treatment. Comparison is not mandated. So, by those criteria montelukast was effective, but I would argue that it was not as effective as albuterol.

**Dr. Lemanske: **In the old days, I believe the FDA considered a significant protective effect as a reduction in the drop in FEV_1 _by 50% or more. However, the published literature usually reports protection if the drop in FEV_1 _goes below 15%. The problem is that while the patient might not drop as far as they were before, they are still dropping. I think that clinicians forget that these drugs don't completely block the drop in FEV_1 _in the majority of patients; and for the competitive athlete, that can be critical.

**Dr. Colice: **Well, you can look at the outcome in a variety of ways: the actual change in FEV_1 _or the percentage of patients that had less than a certain change.

**Dr. Calhoun: **That's exactly right. It's clear that albuterol has a broader efficacy profile, that it prevents EIA in a greater proportion of patients and probably provides a more quantitatively intense protection against bronchospasm. If you look only at those outcomes, albuterol looks to be a better protective agent. But when you look at the broader context, it is less clear. There may be patients for whom montelukast may be a preferred therapy.

**Dr. Colice: **I would agree. As Dr. Calhoun pointed out, if you have a child who has symptomatic asthma and exercise-induced symptoms, montelukast on a regular basis is a very reasonable choice. There clearly are scenarios appropriate for each medication.

**Dr. Luskin: **Also, the convenience issue that was discussed is absolutely real for many people. This is one more example of what's been addressed at this meeting, namely that one size does not fit all.

**Dr. Spector: **Would levalbuterol also show tachyphylaxis?

**Dr. Calhoun: **From the S-albuterol literature, it's reasonably clear that S-albuterol does have adverse effects on airway smooth muscle and probably does increase airway hyperresponsiveness, but at a much higher dose, more than 4 times a day.

**Dr. Colice: **I disagree. I don't think there's any information to suggest that levalbuterol has an advantage in that scenario.

**Dr. Spector: **In view of the discussion about heterogeneity of asthma, wouldn't you postulate the same heterogeneity might be true of EIB? So, if we could sort out that heterogeneity better, we might have a better handle on treatment of EIB for the patient? In clinical trials, if you look at individual data instead of mean data, you see heterogeneity within the group itself.

**Dr. Calhoun: **You're right, there is heterogeneity. There are certainly those patients in whom the release of mast cell mediators occurs and activates an inflammatory cascade. Then there are those patients who don't have much inflammation, so the mediators are probably acting on vascular receptors to cause airway obstruction via perivascular and peribronchial edema. The data are hidden in the means.

**Dr. Colice: **I agree.

**Dr. Oppenheimer: **What are your opinions on the use of cromolyn in EIA?

**Dr. Calhoun: **In the spectrum of efficacy, both in terms of the proportion of people helped and the completeness of efficacy, I'd put cromolyn further down the line. There's more heterogeneity of bronchoprotection with cromolyn, and the degree of effect is variable.

**Dr. Storms: **I disagree. And in light of the few articles that suggest that albuterol may produce a VQ abnormality in the lungs, which could inhibit the ability to improve aerobic performance, wouldn't it better to use a nonbronchodilator?

**Dr. Colice: **It's clear that albuterol has multiple effects, that is not only affects airway smooth muscle tone but also has cardiovascular effects. It's a well-known phenomenon that when someone with asthma comes into the emergency department and gets albuterol, their saturation falls presumably because of worsened VQ matching problems, which probably relate to increased heart rate and cardiac output and other effects on vasculature. But, I think those situations are different because of the substantially higher dose of albuterol that's given and the amount that gets into the systemic circulation.

**Dr. Storms: **If somebody is on combination therapy and then uses albuterol before exercise, is that enough beta-agonist?

**Dr. Colice: **I don't think so.

## Note

Gene Colice, MD, is speaker, consultant, and advisory board member for GlaxoSmithKline (GSK), Pfizer, Lilly, Forest, Almirall, SP, BI, Genetech, and Sepracor; William J. Calhoun, MD, has received grants and research support from Sepracor, Alcon Laboratories, Inc.; he is a consultant for AstraZeneca, Genentech, and Novartis; and the speakers' bureau at AstraZeneca, Genentech, Sepracor, and Merck.
